# Provision of renal-specific nutrition knowledge for changing dietary practice in Bangladeshi hemodialysis patients

**DOI:** 10.1016/j.pecinn.2022.100028

**Published:** 2022-03-14

**Authors:** Tanjina Rahman, Shakil Ahmed, Md. Ruhul Kabir, M. Akhtaruzzaman, Esrat Jahan Mitali, Harun-Ur Rashid, ZulfitriAzuan Mat Daud, Ban-Hock Khor, Deepinder Kaur, Pramod Khosla

**Affiliations:** a Institute of Nutrition and Food Science, University of Dhaka, Bangladesh; b Dept. of Food Technology and Nutrition Science, Noakhali Science and Technology University, Sonapur-3814, Bangladesh; c Kidney Foundation Hospital and Research Institute, Dhaka, Bangladesh; d Dept. of Dietetics, Faculty Medicine and Health Sciences, Universiti Putra Malaysia, Serdang, Selangor 43400, Malaysia; e Faculty of Food Science and Nutrition, Universiti Malaysia Sabah, Malaysia; f Dept. of Nutrition and Food Science, Wayne State University, Detroit, MI, USA

**Keywords:** Nutrition booklet, Bangladeshi phosphorous pyramid, Hemodialysis, Renal-specific nutrition knowledge, Phosphorous to protein ratio

## Abstract

**Objective:**

Studies show that provision of nutrition knowledge help renal patients make informed food choices. This study aimed to evaluate the impact of nutrition knowledge for changing dietary practice among Bangladeshi dialysis patients.

**Methods:**

Following development of a renal-specific nutrition booklet, a pilot study was conducted among 50 hemodialysis patients from a single dialysis setting. Demographic, anthropometric, clinical, biochemical, dietary data, and a 10-item MCQ on renal-specific nutrition information were collected before and 3 months after the provision of the booklet.

**Results:**

52% of the participants were male, 54% had twice weekly dialysis, age 53 ± 12 years, and dialysis vintage was 46 ± 25 months. Serum potassium and phosphorous, dietary potassium, phosphorous, and phosphorous to protein ratio were significantly reduced after the provision of the booklet. Additionally, patients consuming >3 meals/day increased to 66% while adherence to renal-specific cooking method and vegetable preference were significantly increased to 70% and 62%, respectively.

**Conclusion:**

Provision of knowledge via renal-specific nutrition booklet was able to improve patients' dietary practice and enhance their dietary adherence to renal specific recommendations.

**Innovation:**

The booklet was developed using locally available food items in local language and was found beneficial in low-resource settings where overall health care facilities, including nutrition support are limited.

## 1 Introduction

A common trend seen among dialysis patients is an inadequate dietary intake during the transition from non-dialysis to the dialysis period due to imposed dietary restrictions before the initiation of dialysis. Provision of nutrition education showed improvement in controlling the diet-related incidence of hypertension, hyperphosphatemia, hyper/hypokalemia, and protein-energy wasting, thus reducing further deterioration in renal function [1]. Careful and appropriate food selection is crucial to address comorbidities related to chronic kidney disease (CKD). The global prevalence of CKD has been reported to be 11% [2]. In Bangladesh, the prevalence was reported to be 22.48% in a meta-analysis conducted by Banik et al. [3]. Disappointingly, it is not yet possible to estimate the incidence, prevalence, and pattern of kidney diseases in Bangladesh to its full extent [4]. According to hospital statistics, 35,000 to 40,000 people reach end-stage kidney disease each year, and around 40,000 die each year [5]. Therefore, CKD prevention should be integrated with primary health care and kidney health promotion should be part of a health education program that is currently being taken into consideration in developed countries [6]. To our knowledge, no study has been done in Bangladesh to see how better nutrition knowledge affects the health of Bangladeshi patients on hemodialysis who have better nutrition knowledge than before.

Nutritional management in renal disease is challenging for clinicians since the outcome of dialysis depends on the adequacy of both the dialysis treatment and the dietary intake and nutritional status of the patient [7]. Various dietary restrictions are traditionally imposed on dialysis patients, whereas little evidence exists for their benefits [8]. The aims of dietetic intervention among dialysis patients should be to optimise their nutritional status, keep renal biochemistry within safe limits, control blood pressure, blood glucose, and fluid overload, and thus make dietary advice as practical as possible to aid compliance. For this reason, all renal patients should have adequate renal-specific dietetic or nutritional support [9]. Several epidemiological studies have revealed that patients with kidney disease have a poor understanding of their illness and little knowledge of renal-specific diet practices [1,[10], [11], [12], [13]].

In general, poor dietary intake mainly represents inadequate dietary energy and protein intake of a patient, if not specified otherwise. A recent study showed that high intake of both red meat and its processed products was linked to a high risk of CKD, compared to a diet rich in nuts, legumes, and skimmed milk products using a food frequency questionnaire (FFQ) [14]. A high blood urea level may increase protein carbamylation and the production of reactive oxygen species, which leads to oxidative stress, inflammation, endothelial dysfunction, and cardiovascular disease [15]. Restricting dietary protein causes a reduction in urea generation. However, to manage increased protein loss due to ongoing dialysis procedures, the Kidney Disease Outcome of Quality Initiatives (K/DOQI) recommended 1.0 to 1.2 g of protein per kg of body weight per day for a typical hemodialysis patient [16].

In addition to protein, phosphorus homeostasis is compromised in CKD patients. Recent data showed that dietary phosphorus restriction might compromise the need for adequate protein intake. That leads to protein energy wasting among patients with CKD, which ultimately increases both morbidity and mortality among them. Controlling alimentary phosphorus is pretty perplexing in real-life settings. In addition to the amount of dietary phosphorus, the types of phosphorus (organic vs inorganic), sources (animal vs plant foods), and its ratio with dietary protein are also important.

The objectives of this study was to utilize Bangladeshi food composition data [17] to help a group of disadvantageous dialysis patients make informed food choices to improve their adherence to renal-specific diets. Therefore, a pilot study was carried out to determine the impact of a targeted nutrition information dissemination among the dialysis population in a low-resource setting using a 10-item multiple-choice questions (MCQ).

## 2 Methods

**Fig. 1:**
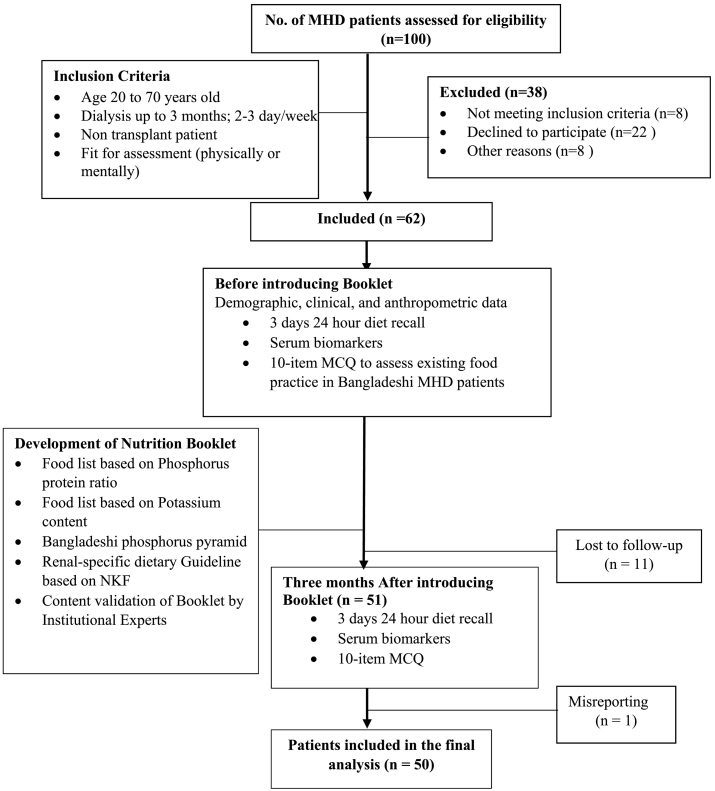
Pilot study flow chart.

A pilot study was conducted to determine whether the provision of renal-specific nutrition knowledge could help in improving the existing dietary practice among Bangladeshi dialysis patients (Fig. 1). At first, a total of 381 local food items were listed based on KDOQI guidelines [18] and the Bangladeshi Food Composition Table [17] in terms of renal-specific nutrients, such as protein, phosphorous, potassium, sodium, and phosphorous to protein ratio. To the best of our knowledge, most Bangladeshi people are not aware of the existence of a phosphorous pyramid. Keeping this in mind, we tried to make a phosphorus pyramid that could be used in this study's nutrition education tool. We used data from the Food Composition Table for Bangladesh [17] to come up with the pyramid.

### 2.1 Development of a nutrition education tool

At the beginning, a table with recommended cut-off points for renal-specific nutrients was made (Table 1). Then, some renal-friendly Bangladeshi food stuffs were listed considering their protein, phosphorus (P), P to protein ratio, potassium (K), and sodium (Na) content (values were available mostly from raw food) that are thought to be the major concerns in the dietary management of patients with chronic kidney disease (*Supplementary Table 1*) [19].

**Table 1:** Recommendations for renal-specific nutrient cut-off points [18].

Nutrient/100 g of Food	Low	Medium	High	Very High
Phosphorus to protein ration (mg/g)	< 12 mg/g	12-15 mg/g	>15 mg/g	
Protein (g)	<20 g	(20-50) g	>50 g	
Phosphorus (mg)	<50 mg	50-150 mg	>150 mg	
Potassium (mg)	<100 mg	101-200 mg	201-300 mg	>300 mg

Here, low means desirable, medium means moderately desirable, high means not desirable and very high means detrimental.

**Fig. 2:**
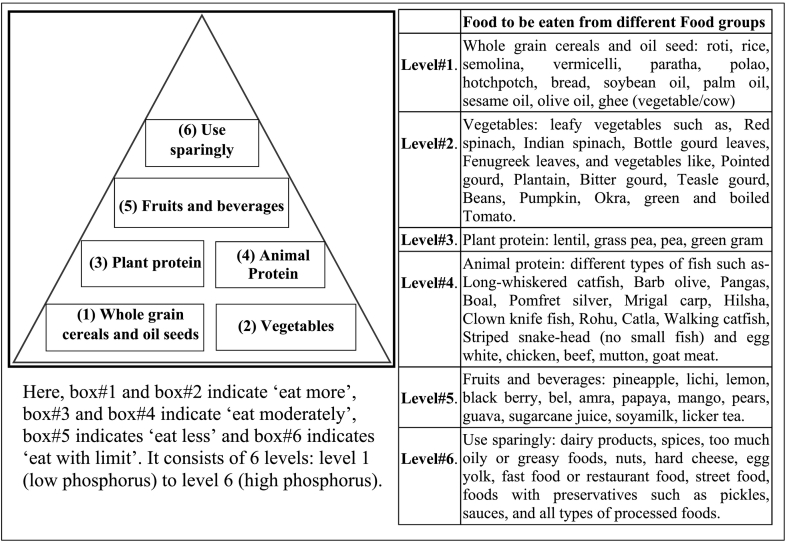
Bangladeshi phosphorous pyramid for hemodialysis patients.

Then, a phosphorus pyramid in accordance with “International Phosphorous pyramid [20]” was made with food items that were found to be good for people in the area from the previous analysis of all 381 food items *(*Fig. 2*)*.

*In*Table 2*,* foods from meat, egg, fish and legumes were listed based on their phosphorous (P) to protein ratio and separated as “eat more” if the ratio is <15 mg of P per gram of protein and “eat less” if the ratio is >15 mg of P per gram of protein.

**Table 2:** List of fish, meat and legumes based on Phosphorous to Protein (P: Pro) Ratio (mg of P per g of Pro) [17].

	Eat More (P:Pro of < 15)	Eat Less (P:Pro > 15)
Fish	Long-whiskered catfish, Pangas, Rohu, Pomfret silver, Hilsha, *(Striped snake-head, Stone roller,Calbasu, Barb Olive, Boal, Croaker, Climbing perch Thai, Giant sea perch, Indian threadfin, Silver carp, dried fish, Clown knife fish, Minnow Large scale razorbelly, Walking catfish, Catla, Gangetic mystus, Giant tiger prawn, Giant river prawn)	Pomfret black, Ganges river sprat, Mola carplet, Gourami, Gangetic ailia, *(Mrigel, Pabda, tilapia, Stinging catfish, Climbing perch indigenous, Bacha, Goby, Bronze feather back, Spotted snakehead, Mullet, Mottled Nandus, Silver needle fish, Pool barb, Indian river shad, Horina Shrimp)
Meat & egg	Egg from organic chicken and meat, mutton, pork, *(goat meat, beef, buffalo meat, pork, duck meat, pigeon meat)	Egg from commercially farmed chicken, Duck egg, cheese, whole cow's milk, sweet yogurt, powdered milk, condensed milk, whey
Pulse & legumes	Lentil, green gram, peas, grass peas	Chickpea/Bengal gram, Black gram

(*) means-foods high in potassium content and it can be reduced by boiling and proper cooking method [17].

Table 3 shows the list of foods from fruits and vegetables in which potassium is present in low, moderate, and high amounts based on the guideline per 100 g of food. Some vegetables, like boiled radishes and cabbage, have less potassium in them than raw radishes and cabbage do. This is because boiling makes them less potassium-rich.

**Table 3:** Amount of potassium per 100 g of Fruits and vegetables [17].

	Less (0-100 mg)	Moderate (101-200 mg)	More (Above 201 mg)
Vegetables	Radish (boil), green papaya (boil), cabbage (boil), okra (boil), carrot (boil)	Cucumber, pointed gourd (boil), green papaya, radish, eggplant (boil), snake gourd, ash gourd, bottle gourd, sponge gourd, cabbage, bitter gourd (boil), teasle gourd, pointed gourd, carrot, plantain (boil), green and ripe tomato, black bean, eggplant, sweet potato (boil)	Onion, green chili, cauliflower boil, broad bean, plantain, peas, drumsticks, pumpkin, beet, cowpea, garlic, sweet potato, potato, colocassia, taro, edd
Greens/leaves	Fenugreek/methi	Indian spinach, red amaranth (boil), green amaranth leaves (boil)	Water spinach, water cress, red amaranth, bottle gourd leaves, potato leaves, raddish leaves, spinach, colocassia leaves
Fruits	Apple (w/o skin)	Jamrul, apple, water-melon, pineapple, Carambola, pear, muskmelon, lichi, orange, pomegranate, black berry, mango (langar), ripe papaya, grape, malta	Mango (fazli), melon, embelic, pomelo, jujube, wood apple, elephant apple, tamarind, dates, banana, jackfruit, custard apple, palmyra palm

Raw vegetables high in potassium content can be reduced through proper cooking method [17].

Then*,* some tips for reducing potassium content from tuberous vegetables through cooking procedure were provided in (*Supplementary Table 2*) [21] with the aim of changing the existing cooking method of those vegetables in the patient pool.

Finally, all this information was incorporated into the booklet (*Supplementary Fig. 1*). In the end, the booklet was checked by the Institute of Nutrition and Food Science at the University of Dhaka for its nutrient analysis and by the KFHRI for its clinical acceptance (*Supplementary Fig. 2*).

### 2.2 Human subjects

Initially, 100 hemodialysis patients were assessed for eligibility at Kidney Foundation Hospital and Research Institute (KFHRI) using a convenient sampling technique [22], of which, 62 patients were included for this study (Fig. 1). Data was collected prior to the introduction of the nutrition booklet (September 2019), and three months later (December 2019), 11 patients dropped out and 1 patient was found to be a mis-reporter. 50 patients were included for statistical analysis. A protocol for this study was approved by the ethical board of KFHRI in Dhaka, Bangladesh. Inclusion criteria were hemodialysis patients who underwent either twice or thrice weekly dialysis for at least 3 months and who gave informed consent for continued participation in the study 3 months post-introducing the booklet.

Before the provision of the booklet to each patient, a one-page 10-item multiple choice question (MCQ) based on simple renal-specific nutrition information and food practice was administered to get a brief picture of their existing food practice. Then, the same question was asked after 3 months of the same set of patients to look for any changes due to the provision of this booklet (*Supplementary Table 3*). This 10-item MCQ was formulated based on inputs from nephrologists and nutritionists from the hospital, and the internal consistency reliability for the 10-tem MCQ was judged based on the average inter-item correlation (AIC) analysis and calculating Cronbach's alpha. The AIC for the 10-item MCQ used in this study is 0.58 (0.399-0.961) and Cronbach's alpha is 0.93. Both the obtained AIC and alpha value proved to have adequate internal consistency for the 10-item MCQ [23]. The purpose of this MCQ test was to help us get a rough idea about the level of existing nutrition knowledge and food practices among dialysis patients and the extent to which the targeted nutrition information related booklet had an impact on their current level of knowledge and food practices.

Additionally, both biochemical and dietary data for all patients were also collected and analyzed before and after the provision of the booklet. The dietary reports were gathered using the 3-day dietary recall (3DDR) approach, with 24-h diet recalls gathered for a dialysis day, a non-dialysis day, and a weekend day [24]. Data from the 3DDRs were analyzed using the computer software Nutritionist Pro™ (Axxya Systems LLC, Stafford, TX, USA) by referencing the Bangladesh Food Composition Table (FCT), [25,26] and Standard food recipes were constructed when the food item was not available in any other databases, as previously reported [27]. The Goldberg cut-off values were implemented to assess misreporting or implausible diet records based on a physical activity level (PAL) of 1.0, which indicates moderate-low physical activity level, and compared with the ratio of Energy Intake (EI) to Basal Metabolic Rate (BMR), where an index value of <0.8 was considered as under-reporting, 0.8–2.4 was considered as acceptable reporting, and EI: BMR >2.4 as over-reporting [28]. The patients BMR was calculated by using the Harris–Benedict eq. [29].

### 2.3 Statistical analysis

Descriptive statistics of continuous variables were presented as means with standard deviation or frequency/percentage (n, %), depending on the distribution of variables. Categorical variables were presented as distributions (frequency and percentage). A *p*-value of less than 0.05 was considered statistically significant for both the one-way ANOVA (continuous variables) and Pearson's Chi-square test, followed by the Bonferroni post hoc test (categorical variables). For statistical analyses, SPSS version 26 (IBM, Chicago, IL) was used (IBM, Chicago, IL).

### 2.4 Potential pitfalls

The “one-page 10-item MCQ” that was used to measure “existing nutrition knowledge and food practice” in patients was made and tested in the study area by nephrologists and nutritionists who were available. It was made and tested in the study area by nephrologists and nutritionists who were available.

### 2.5 Ethical concideration

This study received ethical approval from the ethical boards of Kidney Foundation Hospital and Research Institute in Dhaka, Bangladesh (KFHRI). KFHRI and the Institute of Nutrition and Food Science at the University of Dhaka also vetted and approved the clinical and nutritional information of the booklet to be used as a patient educational tool in this study.

## 3 Results

Table 4 shows the demographic characteristics, medical history, and anthropometrics of these 50 patients: 26 were male (52%), and 27 (54%) of them were undergoing twice-weekly dialysis. The mean age was 53 ± 12 years, of which only 8% were younger adults (18-35 years), 50% were middle-aged (36-55 years) and 42% were older adults (> 55 years) [30]. The duration of dialysis was 3.8 ± 0.3 h, and the dialysis vintage, 46 ± 25 months. The causes of End-stage kidney disease (ESKD) were reported to be 38% due to hypertension (HTN), 30% due to diabetes mellitus and/or hypertension (DM/HTN), 20% due to HTN or chronic glomerulonephritis (HTN/CGN), and 13% due to other reasons 2 patients due to acute polycystic kidney disease (APKD) and 4 due to unknown reasons.

**Table 4:** Demographics and medical history of patients (*n* = 50) who used the “Nutrition Booklet”.

Demographics	All (n = 50)
Gender, male, n (%)	26 (52%)
Age (Years)	53 ± 12
Young adult (18-35)	4 (8%)
Middle-aged (36-55)	25 (50%)
Older adult > 55	21 (42%)
Duration of dialysis (hours)	3.8 ± 0.3
Dialysis vintage (months)	46 ± 25

Dialysis frequency, n (%)	
Thrice a week	23 (46%)
Twice a week	27 (54%)

Causes of ESRD, n (%)	
HTN	18 (38%)
HTN and DN	15 (30%)
HTN and CGN	10 (20%)
Other (2 APKD, 4 UK)	6 (13%)
Missing data	1 (2%)

Anthropometrics	
Height (cm)	160 ± 10
Body Weight (Kg)	64 ± 12
BMI (kg/m^2^)	25.2 ± 5.2

BMI category, n (%)	
Under-weight/usual weight/over-weight/obese	4 (8%)/22 (46%)/16 (33%)/6 (13%)
MAC (cm)	28.9 ± 4.4
TSF (mm)	17.0 ± 7.7
MAMC (cm)	23.5 ± 2.8
cAMA (cm^2^)	35.5 ± 11.2
HGS (kg)	21 ± 9

Data were collected from 50 patients (24 females and 26 males) at Kidney Foundation Bangladesh. Values are mean ± SD and n or %. HTN: Hypertension, DN: Diabetic nephropathy, CGN: Chronic glomerulonephritis, other: APKD, kidney stone, Unknown, postpartum complication; genetic, ESRD: End-stage renal disease. BMI: body mass index, BMI category is based on WHO guideline [31]. MAC: mid-arm circumference, TSF: triceps skin-fold thickness, MAMC: mid-arm muscle circumference, cAMA: corrected arm-muscle area, HGS: hand grip strength.

Data were analyzed using *one-way ANOVA* for continuous variables (Table 5 *and* Table 6) and *Pearson's chi-square test followed by bonferroni* post hoc *test* (Table 7) for categorical variables to see if there is any significant differences following introduction of “nutrition booklet”.

**Table 5:** Findings of biochemical variables before and 3 months after the provision of the “Nutrition Booklet”.

Biochemical parameters	Before	After	*P-value*	Reference value
*TIBC (mg/l)*	244 ± 56	253 ± 70	*0.521*	300-400 [32]
*URR (%)*	65 ± 7	64 ± 7	*0.268*	≥65 [33]
*Na (mEq/l)*	134 ± 3	135 ± 3	*0.308*	135-146 [34]
*K (mEq/l)*	5.2 ± 0.8	4.8 ± 0.5	***0.008***	3.5-5.3 [35]
*Phosphorous (mEq/l)*	5.8 ± 1.7	5.0 ± 1.5	***0.024***	
*Albumin g/dL)*	3.8 ± 0.3	3.8 ± 0.3	*0.873*	3.8-5.0 [36]
*Ferritin (ng/ml)*	541 ± 451	455 ± 392	*0.390*	5-275 [37,38]
*Ferritin>2000 (ng/ml)*	9 (19%)	14 (29%)		
*CRP (mg/L)*	14 ± 20	12 ± 17	*0.71*	

Data were collected from 50 patients (24 females and 26 males) at Kidney Foundation Hospital and Research Institute. Values are mean ± SD (n). BMI: Body mass index, TIBC: total iron-binding capacity, URR%: Urea reduction rate%, Na: sodium, K: potassium, P: phosphorous, CRP: C-Reactive protein. One-way ANOVA was done and a p-value<0.05 was considered statistically significant.

**Table 6:** Findings of dietary intakes from acceptable reporters before and 3 months after the provision of the “Nutrition Booklet”.

Dietary intakes	Before (41)	After (44)	P-value	K/DOQI Guideline
*ADAT Score*	2.9 ± 0.6	2.7 ± 0.8	*0.259*	
*Energy (calorie)*	1534 ± 386	1601 ± 249	*0.343*	*Based on BW*
*DEI (kcal/kg-BW/day)*	25 ± 6	26 ± 7	*0.392*	*25-35*
*Protein (g)*	58 ± 16	62 ± 12	*0.184*	*Based on BW*
*DPI (g/kg-BW/day)*	0.9 ± 0.3	1.0 ± 0.3	*0.273*	*1.0-1.2*
*Carbohydrates (g)*	216 ± 55	218 ± 46	*0.823*	*Based on BW*
*Fat (g)*	46 ± 14	50 ± 13	*0.128*	*Based on BW*
*% calorie from Carbohydrates*	57 ± 5	54 ± 6	***0.041***	
*% calorie from protein*	15 ± 2	17 ± 3	***0.008***	
*% calorie from Fat*	27 ± 4	28 ± 6	*0.157*	
*Sodium (mg)*	2008 ± 745	2036 ± 576	*0.847*	*<2400*
*Potassium (mg)*	1667 ± 386	1410 ± 332	***0.001***	*Individualized*
*Phosphorous (mg)*	855 ± 243	755 ± 160	***0.026***	*1000*
*P/kg BW/day*	13.7 ± 3.9	12.1 ± 3.6	***0.052***	*10-17*
*P to protein ratio*	14.9 ± 1.7	12.6 ± 2.9	***0.000***	*<12*
*Calcium (mg)*	414 ± 202	429 ± 237	*0.756*	*<1000*
*Iron (mg)*	13 ± 6	14 ± 4	*0.280*	*Individualized*
*Magnesium (mg)*	260 ± 66	256 ± 56	*0.760*	*200-300*

Dietary data were collected from 50 patients (24 females and 26 males) at Kidney Foundation Hospital and Research Institute. Values are mean ± SD (n). ADAT [39]: Appetite and diet analysis tool. Scale: 1 = very good, 2 = good, 3 = fair, 4 = poor, and 5 = very poor. DEI: dietary energy intake, DPI: dietary protein intake, P: Phosphorous, BW: body weight, K/DOQI: kidney disease outcome and quality initiatives. One-way ANOVA was done and a p-value<0.05 was considered statistically significant.

**Table 7:** Outcomes in changing existing food practice after using the “Nutrition Booklet”.

10-item MCQ	Parameters	Before, n (%)	After, n (%)	*P-value*
No. of meal/day	*≤3 meals*	35 (70)	17 (34)	***0.001***
	*>3 meals*	15 (30)	33 (66)	
Follow cooking method	*Yes*	13 (26)	35 (70)	***0.000***
	*No*	37 (74)	15 (30)	
Follow fluid guideline	*Yes*	27 (54)	34 (68)	*0.151*
	*No*	23 (46)	16 (32)	
Daily Egg intake	*Yes*	30 (60)	34 (68)	*0.405*
	*No*	20 (40)	16 (32)	
Egg Intake preference	*Both whole and egg white*	31 (62)	20 (40)	*0.056*
	*Egg white*	19 (37)	30 (60)	
Pulse preference	*All types*	35 (70)	22 (44)	*0.019*
	*Healthy pulse*	15 (30)	28 (56)	
Meat preference	*Only white meat*	29 (58)	26 (52)	*0.546*
	*Both red and white meat*	21 (42)	24 (48)	
Fish preference	*Only small fish*	34 (68)	21 (42)	*0.019*
	*Both big and small fish*	16 (32)	29 (58)	
Vegetable intake preference	*Yes*	16 (32)	31 (62)	***0.005***
	*No*	34 (68)	19 (38)	
Fruit intake preference	*Yes*	18 (36)	31 (62)	*0.019*
	*No*	32 (64)	19 (38)	

Data were collected from 50 dialysis patients (26 males and 24 females) who were previously identified as acceptable reporters, at two time points- before and after providing “Nutrition Booklet” using a “10-item multiple choice questions (MCQ)” from Kidney Foundation Hospital and Research Institute. Values are in percentage (%). Adjusted p-value was calculated using Bonferroni post hoc test where the *p*-value<0.013 indicates statistically significant difference after using Pearson's Chi square test.

In Table 5, Biochemical parameters revealed significant differences in terms of serum potassium 5.2 ± 0.8 mEq/l (pre) and 4.8 ± 0.5 mEq/l (post) and phosphorous 5.8 ± 1.7 mEq/l (pre) and 5.0 ± 1.5 mEq/l (post). Total‑iron binding capacity (TIBC) was slightly higher: 244 ± 56 μg/l (pre) and 253 ± 70 μg/l (post), and CRP slightly lower 14 ± 20 mg/l (pre) and 12 ± 17 mg/l (post) (*p* *>* *0.05*). However, no significant differences in Urea Reduction Ratio (URR), serum sodium, ferritin, and serum albumin level were observed. During booklet introduction, 9 patients had ferritin levels of >2000 ng/ml and after 3 months, 14 patients had ferritin levels of >2000 ng/ml.

*In*Table 6*,* dietary data were analyzed only for acceptable reporters before and after the booklet was introduced, and we found no significant differences in terms of dietary energy intake (DEI), dietary protein intake (DPI), carbohydrate or fat intake, sodium, calcium, iron, or magnesium intake, which was slightly increased after the booklet was provided. However, while comparing with guidelines provided by K/DOQI for hemodialysis patients [16], both DEI and DPI intakes were found lower among the study cohort, before and after introducing the nutrition booklet. Therefore, significant differences were observed in terms of % calorie intake from carbohydrates: 57 ± 5 (pre) and 54 ± 6 (post) and % calorie intake from protein intake 15 ± 2 (pre) and 17 ± 3 (post) *(p* *<* *0.05)*, and for dietary potassium 1667 ± 386 mg (pre) vs 1410 ± 332 mg (post), dietary phosphorous 855 ± 243 mg (pre) vs 755 ± 160 mg (post), and phosphorous to protein ratio- 14.9 ± 1.7 (pre) and 12.6 ± 2.9 (post) mg P/g protein. In terms of P intake per kg body weight/day, a slightly significant difference was also observed, 13.7 ± 3.9 (pre) and 12.1 ± 3.6 (post) (*P* *=* *0.052*).

In Table 7, provision of the “Nutrition Booklet” specifically for dialysis patients showed a crude picture of improvements in patients' food intake patterns and adherence towards renal-specific diet practice using the 10-items MCQ among 50 MHD patients, where the responses from study patients were analyzed and significant differences were observed between pre and 3 months post booklet provision: frequency of meal eaten by patients increased >3 meals a day from 15 (30%) patients (pre) vs 33 (66%) patients (post). The chi-square test was statistically significant, *χ*^*2*^ (1, *N* = 50) = 12.98, *p* < 0.013, with *Phi (φ)* coefficient of 0.36. Adherence to specific cooking method: 13 (26%) (pre) and 35 (70%) (post) [*χ*^*2*^ (1, N = 50) = 19.39, *p* < 0.013, with *Phi (φ)* coefficient of 0.44], adherence to fluid guideline: 27 (54%) (pre) and 34 (68%) (post) [*χ*^*2*^ (1, N = 50) = 2.06, *p* = 0.151, with *Phi (φ)* coefficient of 0.14], based on knowledge about potassium content indifferent food items, adherence to vegeables preferences: 16 (32%) (pre) vs 31 (62%) (post) [*χ*^*2*^ (1, *N* = 50) = 9.03, *p* < 0.013, with *Phi (φ)* coefficient of 0.301] and fruits-18 (36%) (pre) vs 31 (62%).(post) [*χ*^*2*^ (1, *N* = 50) = 6.76, *p* = 0.019 > 0.013, with *Phi (φ)* coefficient of 0.26]. Egg is an inexpensive source of protein with high-biological value and dialysis patients require optimum protein, especially with high biological value to cope up with their increased requirement and egg could be a suitable option for them as no adverse effect was found with egg consumption and kidney disease progression till now [40]. Around 30 (60%) patients were reported to take egg on a daily basis before introducing the booklet and after 3 months, approximately, 34 (68%) patients were reported to eat egg on daily basis [*χ*^*2*^ (1, *N* = 50) = 0.694, *p* = 0.056 > 0.013, with *Phi (φ)* coefficient of 0.08]. Then to assess booklets' efficacy, the questionnaire used in this study revealed that consumption of egg white: 19 (38%) (pre) vs 30 (60%)% (post) [*χ*^*2*^ (1, N = 50) = 4.84, *p* = 0.056 > 0.013, with *Phi (φ)* coefficient of 0.22]. Therefore, no significant difference was found in terms of pulse consumption (a slight shift towards a healthy preference and preferences in fish consumption) [*χ*^*2*^ (1, N = 50) = 6.76, *p* = 0.019 > 0.013, with *Phi (φ)* coefficient of 0.26]-towards a healthy choice were observed among patients. Meat consumption preference also did not show any significant changes befre or after introducing the booklet.

## 4 Discussion and conclusion

### 4.1 Discussion

Choices about what to eat and drink while on hemodialysis can make a difference in how they feel and can make their treatments work better. The Nutrition Booklet, “Necessary Nutrition Information for the Better Health of Bangladeshi Dialysis Patients,” was developed to assist HD patients to know, learn, and practice dietary intake. It is important for patients to recognize the importance of the phosphate to protein ratio (P: Pro) in their diet for achieving a diet low in phosphate load. Implementation of some healthy cooking methods can also help in reducing phosphate content during meal preparation. Eating too much potassium can be dangerous for the heart and may even cause death. Food selection and cooking methods may also play a role in limiting dietary potassium levels. Therefore, the nutrition booklet that was developed in this study considering cultural and linguistic barriers and made available as a user-friendly education tool, is more convenient for the patients and greatly helps them in their informed food choices.

Studies previously done in this field showed that effective renal-specific knowledge among patients could reduce the deterioration of renal function among patients at early stages of renal disease [41]. Studies also showed that patients with chronic diseases who had nutrition counseling were shown to have positive outcomes compared to patients who had not received any counseling by analyzing height, weight, recent laboratory data, past medical history, and 24-h diet recall both before and after nutrition counseling [42]. Studies have also shown that interactive educational interventions can help patients with CKD learn more about their disease, take better care of themselves, and have better medical outcomes [43].

The attempts that we made through this pilot study significantly improved some clinical outcomes of the study patients, such as their serum phosphate and serum potassium levels. Additionally, patients' adherence towards following dietary and fluid guidelines also improved. However, this intervention should be done in a more organized way within a large group of dialysis patients, involving a multidisciplinary team in order to get the actual impact of the booklet. A renal-specific nutrition education workshop can be organized involving patients from multiple dialysis centers throughout the country, and a qualitative component could be included during the usability testing of the booklet. The booklet could be modified by providing nutrient composition based on serving size of locally consumed food items (considering seasonal variation) by implementing visual aids. Promotion of healthy lifestyles such as moderation in salt use, limiting intakes of high sugar or fatty foods, processed and fast foods, and identification of inorganic and organic phosphate content in local foods should take place in every part of the country. Renal-specific food labelling should be introduced to help consumers choose what they should buy. Moreover, food adulteration should be monitored more strictly.

### 4.2 Innovation

CKD patients usually have limited knowledge about their illness. Provision of renal-specific nutrition knowledge might play an important role in improving their health and nutritional status. This may be especially important in resource-poor settings where nutritional support is a low priority among healthcare providers. The goals of nutrition therapy for dialysis patients are to provide them with an attractive and palatable diet, improve nutritional status while controlling any complication related to CKD such as fluid overload and abnormal serum electrolytes. Boiling helps to remove excess minerals (phosphorus and potassium) from food of both plant and animal origin. According to studies, boiling removes 51% of the P from vegetables, 48% from legumes, and 38% from meat [44]. Considering loss of phosphorus via different cooking methods, phosphorus pyramids could be used as an important tool for educating patients with chronic kidney disease.

Compared with other educational interventions, the most innovative part of using this renal-specific nutrition booklet as a tool to improve dietary practice among patients is the utilization of the local food composition table to get a list of locally consumed and indigenous food items based on international guidelines and then make it available in the local language, Bangla. There were patients who were not familiar with foreign languages (e.g., English) but could read the local language. Thus, it becomes a handy and effective tool for patients to make informed food choices, follow appropriate cooking practices, and move towards a healthy eating habit, which in turn has a positive impact on their overall health status while on dialysis.

### 4.3 Conclusion

A renal-specific nutrition booklet was developed exclusively for Bangladeshi dialysis patients and made feasible for use in practice as an educational tool to improve their selection of food items as well as their adherence to renal-specific diet practice. Only big city hospitals have dietitians in Bangladesh who provide nutrition and food-related advice to all patients in general. There is no registered renal dietitian in Bangladesh and patients are often confused about what food to eat because of a lack of knowledge about the different types of food available in the country. They either didn't choose any or didn't pick any at all. Thus, their food intake becomes inappropriate, which leads them to the vicious cycle of malnutrition and further complicates their health. Future research is needed to observe the actual impact of the provision of renal-specific nutrition knowledge in the Bangladeshi dialysis community by implementing more traditionally and linguistically personalized education tools and, thus, to improve the dietary intake of hemodialysis patients.

## Authors' contributions

This work was carried out in collaboration among all authors. Authors PK, TR, and HR designed the study, TR, SA, HR, and PK wrote the protocol and the first draft of the manuscript. Authors TR, ZAMD, BHK, and SA performed data collection for the study. Authors EJM, SA, MA and HR aid in validation of educational tool and 10-item MCQ. Authors TR, SA, and MRK managed the analyses of the study. Authors TR, MA, ZAMD, MRK, DK, EJM, and SA managed the literature searches. All authors read and approved the final manuscript.

## Conflict of interests

The authors declare that they have no competing interests.

## Sources of financial support

This research did not receive any specific grant from funding agencies in the public, commercial, or not-for-profit sectors.

## Data availability

Data will be available upon reasonable request.

## Appendix A Supplementary data

Supplementary material Image 1
