# Supplementary material for: Provision of renal-specific nutrition knowledge for changing dietary practice in Bangladeshi hemodialysis patients
Source: PEC Innov. 2022 Mar 14;1:100028. doi: 10.1016/j.pecinn.2022.100028 (PMC10194273; doi:10.1016/j.pecinn.2022.100028)

**Supplementary Table 1.** A snapshot of renal-specific nutrient analysis using Food Composition Table, Bangladesh for some renal-friendly Bangladeshi food stuffs.

| Renal- friendly Food Group | English Name | Protein  (g) | P  (mg) | P: Pro, (mg/g) | K (mg) | Na (mg) |
| --- | --- | --- | --- | --- | --- | --- |
| Beverages | Sugar cane juice | 0.7 | 6.0 | 8.6 | 25.0 | 7.0 |
| Beverages | Jaggery liquid, date palm | 0.3 | 15.0 | ^b^50.0 |  |  |
| Cereals | Vermicelli, boiled | 3.9 | 38.0 | 9.7 | 49.0 | 4.0 |
| Cereals | Semolina, wheat, raw | 10.9 | ^a^105.0 | 9.6 | ^a^158.0 | 5.0 |
| Cereals | Vermicelli, wheat, raw | 8.9 | ^a^92.0 | 10.3 | ^a^140.0 | 8.0 |
| Cereals | Rice flakes, water soaked | 2.0 | 39.0 | ^b^19.5 | 45.0 | 1.0 |
| Fish | Pangas, w/o bones, raw | 15.9 | ^a^130.0 | 8.2 | ^a^169.0 | 46.0 |
| Fish | Boal, w/o bones, raw | 15.4 | ^a^134.0 | 8.7 | ^a^146.0 | 63.0 |
| Fish | Calabash, w/o bones, raw | 17.0 | ^a^141.0 | 8.3 | ^b^287.0 | 100.0 |
| Fish | Prawn, raw | 18.2 | ^a^133.0 | 7.3 | ^c^355.0 | 93.0 |
| Fish | Stripped snakehead, raw | 17.7 | ^a^130.0 | 7.3 | ^c^362.0 | 50.0 |
| Fish | Prawn, raw | 17.6 | ^a^132.0 | 7.5 | ^c^352.0 | 92.0 |
| Fish | Prawn, raw | 18.8 | ^a^141.0 | 7.5 | ^c^375.0 | 98.0 |
| Fish | Stone roller, raw | 15.3 | ^a^124.0 | 8.1 | ^c^834.0 | 35.0 |
| Fish | Giant tiger prawn, raw | 16.5 | ^a^141.0 | 8.5 | ^c^423.0 | 117.0 |
| Fruits | Hog plum, raw | 1.1 | 11.0 | 10.0 | ^a^175.0 | 1.0 |
| Fruits | Pineapple, ripe, raw | 1.0 | 9.0 | 9.0 | ^a^175.0 | 13.0 |
| Fruits | Pineapple, ripe, raw | 0.8 | 7.0 | 8.8 | ^a^122.0 | 42.0 |
| Fruits | Pineapple ripe, raw | 0.8 | 7.0 | 8.8 | ^a^122.0 | 42.0 |
| Fruits | Palmyra palm, raw | 0.6 | 20.0 | ^b^33.3 |  |  |
| Fruits | Bullocks Heart, ripe, raw | 1.4 | 10.0 | 7.1 | ^c^495.0 | 6.0 |
| Greens or leaves | Alligator weed, raw | 4.9 | 46.0 | 9.4 |  |  |
| Greens or leaves | Amaranth leaves, red, boiled, w/o salt | 5.3 | 34.0 | 6.4 | ^a^154.0 | 53.0 |
| Greens or leaves | Indian spinach, boiled, w/o salt | 3.1 | 37.0 | 11.9 | ^a^123.0 | 69.0 |
| Greens or leaves | Fenugreek leaves, raw | 4.4 | ^a^51.0 | 11.6 | 31.0 | 76.0 |
| Greens or leaves | Agathi, raw | 8.4 | ^a^80.0 | 9.5 |  |  |
| Greens or leaves | Amaranth leaves, red, raw | 4.5 | 32.0 | 7.1 | ^b^261.0 | 59.0 |
| Greens or leaves | Bottle gourd leaves, raw | 2.5 | 28.0 | 11.2 | ^b^276.0 | 41.0 |
| Greens or leaves | Cassava leaves, raw | 4.7 | 36.0 | 7.7 | ^c^303.0 | 22.0 |
| Greens or leaves | Bengal dayflower, leaves, | 2.0 | 19.0 | 9.5 | ^c^473.0 | 21.0 |
| Greens or leaves | Colocasia leaves, green | 4.0 | 40.0 | 10.0 | ^c^764.0 | 47.0 |
| Milk | Milk, human, colostrum | 2.0 | 14.0 | 7.0 | 70.0 | 47.0 |
| Meat | Lamb/mutton, meat, moderately fat, raw | 18.5 | ^a^150.0 | 8.1 | ^a^136.0 | 41.0 |
| Milk | Milk, human, mature | 1.2 | 15.0 | ^b^12.5 | 56.0 | 16.0 |
| Oils and Seeds | Ghee, vegetables |  | trace |  | 1.0 | 1.0 |
| Oils and Seeds | Ghee, cow |  | trace |  | 1.0 | 2.0 |
| Pulses | Lentil, boiled, w/o salt | 13.6 | ^a^115.0 | 8.5 | ^b^234.0 | 16.0 |
| Spices | Lemon peel, raw | 1.6 | 12.0 | 7.5 | ^a^160.0 | 6.0 |
| Spices | Coriander leaves, raw | 3.3 | 30.0 | 9.1 | ^c^396.0 | 58.0 |
| Vegetables | Gourd, pointed, raw | 2.0 | 18.0 | 9.0 | ^a^148.0 | 28.0 |
| Vegetables | Gourd, bitter, boiled | 2.3 | 20.0 | 8.7 | ^a^141.0 | 33.0 |
| Vegetables | Gourd, bitter, raw | 2.1 | 20.0 | 9.5 | ^a^182.0 | 36.0 |
| Vegetables | Gourd, pointed, boiled | 2.3 | 18.0 | 7.8 | ^a^115.0 | 26.0 |
| Vegetables | Bean, scarlet, runner | 3.9 | 34.0 | 8.7 | ^b^220.0 | trace |
| Vegetables | Plantain, raw | 2.0 | 21.0 | 10.5 | ^b^242.0 | 4.0 |
| Vegetables | Chili green, raw | 2.8 | 30.0 | 10.7 | ^b^282.0 | 12.0 |
| Vegetables | Carrot, boiled, w/o salt | 1.1 | 39.0 | ^b^35.5 | 81.0 | 40.0 |
| Vegetables | Okra, boiled, w/o salt | 1.7 | 21.0 | ^b^12.4 | 99.0 | 24.0 |
| Vegetables | Amaranth, stem, raw | 0.9 | 30.0 | ^b^33.3 |  |  |
| Vegetables | Pumpkin, boiled, w/o salt | 2.2 | 23.0 | 10.5 | ^c^371.0 | 13.0 |

Here, the column for ‘Protein (g)’ and ‘Sodium or Na (mg)’ and all values that have no ‘superscript’ are in desirable or safe range, superscript ^a^ indicates ‘moderately desirable’, ^b^ indicates ‘not desirable’ and ^c^ indicates ‘detrimental’. P: Phosphorous, K: Potassium, Na: Sodium, Pro: Protein.

**Supplementary Table 2.** How to Leach Potassium from tuberous Root Vegetables.

|  | Cooking procedure |
| --- | --- |
| #1 | Wash and peel the vegetables and slice the vegetables into thin slices |
| #2 | Place the sliced vegetables in room temperature water. Use two times the amount of water to the amount of vegetable. |
| #3 | Bring the water to a boil and drain off the water and add fresh, room temperature water. |
| #4 | Use two times the amount of water to the portion of vegetables, bring the water to a boil again and cook until the vegetable is soft and tender. |

Excess mineral content can be reduced following this cooking procedure

**Supplementary Table 3:** 10-item MCQ to assess existing food practice for Bangladeshi hemodialysis patients

| 1. How many times a day do you take food? |
| --- |
| 1. ≤ 3 meals/day |
| 1. > 3 meals/day |
| 1. Do you follow any specific cooking method while preparing food? |
| 1. Yes |
| 1. No |
| 1. Do you follow any fluid guideline? |
| 1. Yes |
| 1. No |
| 1. Do you eat egg daily? |
| 1. Yes |
| 1. No |
| 1. In which way you prefer to eat egg? |
| 1. Both whole egg and egg white |
| 1. Egg-white |
| 1. What is your comment on the following: (pulse/legumes)? |
| 1. Healthy pulse |
| 1. All types of pulse/legumes |
| 1. What is your comment on the following: (meat)? |
| 1. Only white meat |
| 1. Both white and red meat |
| 1. What is your comment on the following: (fish)? |
| 1. Only small fish |
| 1. Both big and small fish |
| 1. Preference in case of consuming any vegetables? |
| 1. Yes |
| 1. No |
| 1. Preference in case of consuming any fruits? |
| 1. Yes |
| 1. No |
| Subject Code: Completed by: Date: |

**Supplementary Figure 1.** Snapshot of the Nutrition Booklet, “Necessary Nutrition Information for the better heath of Bangladeshi Dialysis Patients”
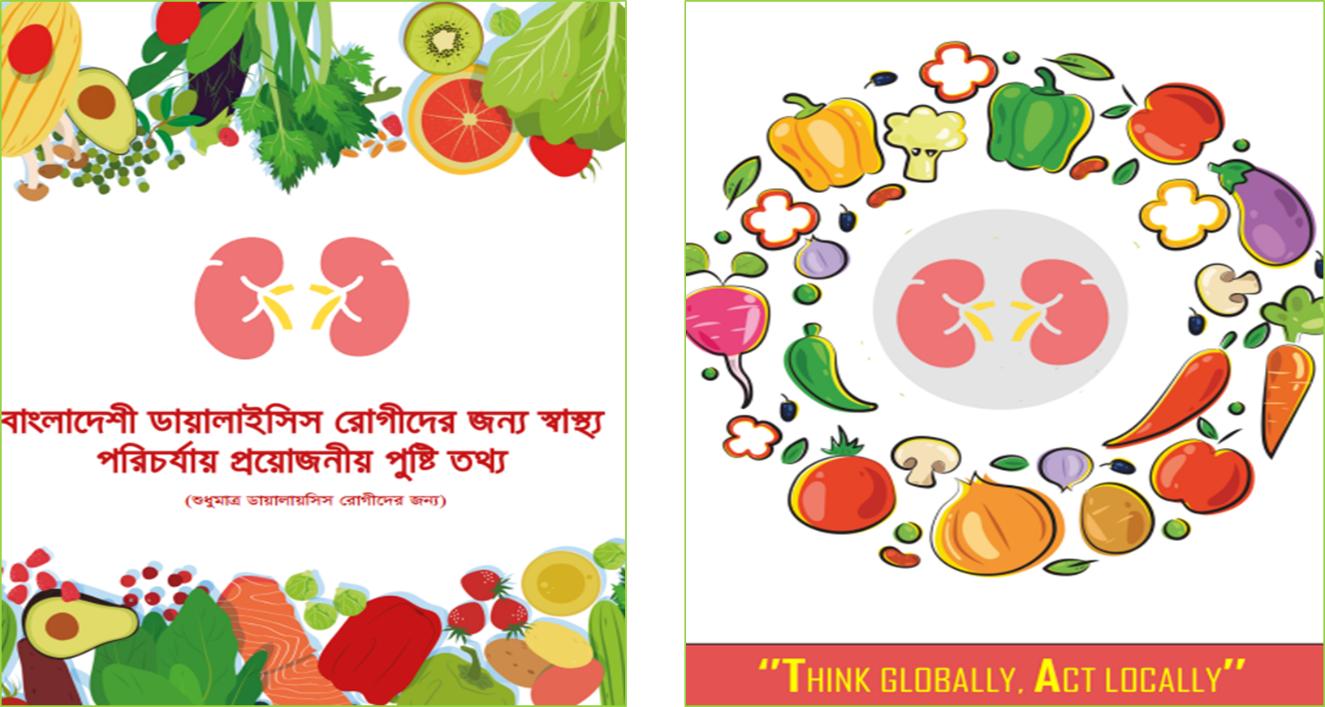


| **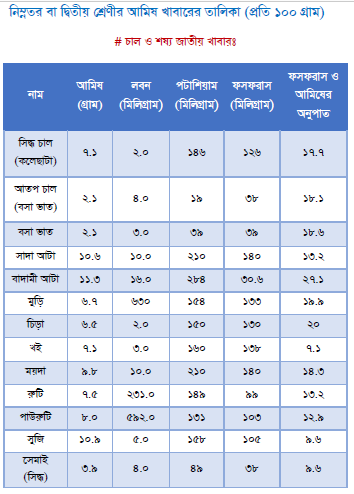** | **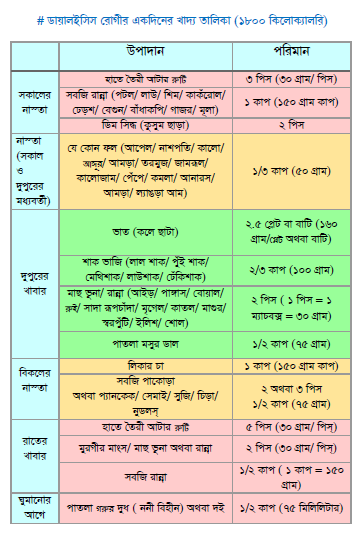** |
| --- | --- |

**Supplementary Figure 2.** Booklet Endorsed by Kidney Foundation, Bangladesh, and University of Dhaka.


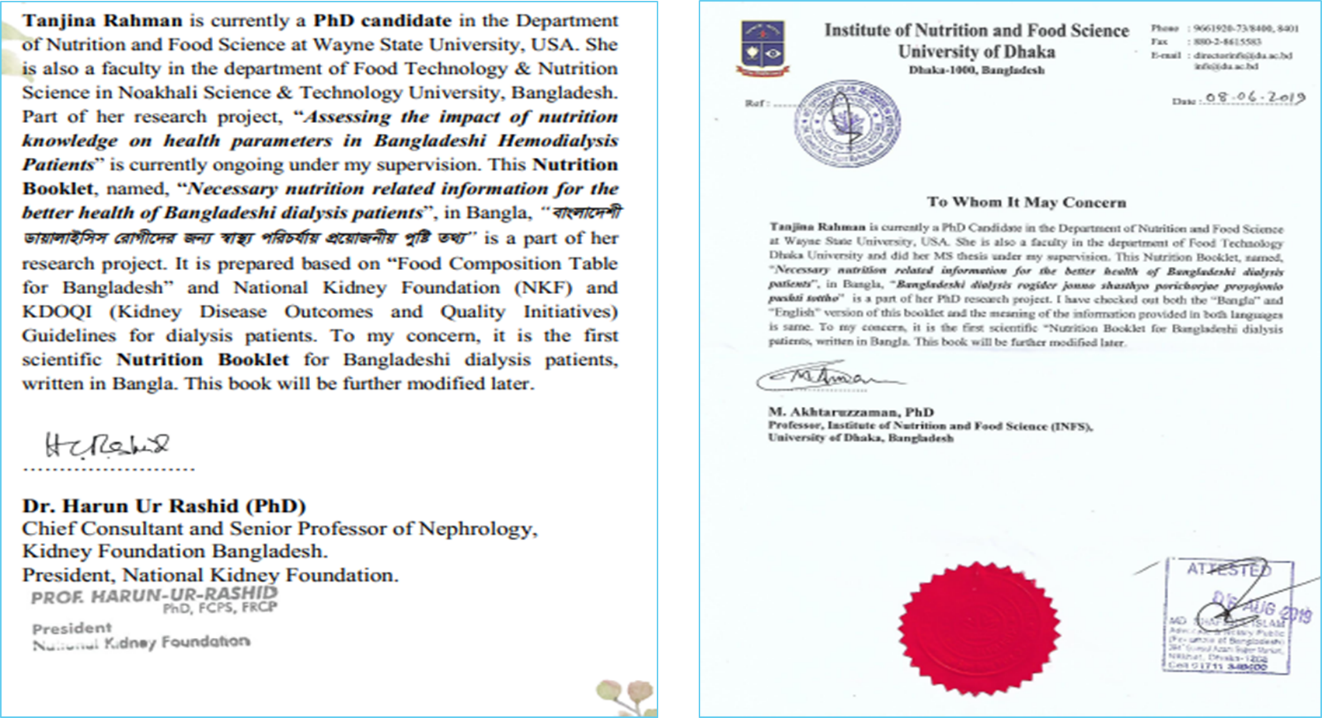

Supplement: Supplementary file 1 — Supplementary material [file mmc1.docx]
